# Development of recombinant antibodies for reliable and sensitive food allergen detection

**DOI:** 10.1186/2045-7022-5-S3-P52

**Published:** 2015-03-30

**Authors:** Jaana Haka, Merja Niemi, Kristiina Iljin, Vanga Siva Reddy, Kristiina Takkinen, Marja-Leena Laukkanen

**Affiliations:** 1 VTT Technical Research Centre of Finland, Espoo, Finland; 2 Department of Chemistry, University of Eastern Finland, Joensuu, Finland; 3 International Centre for Genetic Engineering and Biotechnology, New Delhi, India

## 

Around 4 % of the population suffer from IgE-mediated food allergies in Western countries and the number of food-allergenic people is increasing. Individuals with certain pollen allergies may also suffer from a sensitisation to proteins in the foods. As an example a person sensitised to the major birch pollen allergen, Bet v 1, is often sensitised to its homologues, such as the major allergens of apple, Mal d 1, and celery, Api g 1, as well. Development of diagnostic tools for the reliable, sensitive and quick detection of allergens present in various food products are essential for allergic persons to prevent the consumption of substances causing mild and even life-threatening immune responses. Traditionally the diagnostics of allergens are based on the use of human IgE serum pools isolated from allergic patients. The use of monoclonal antibodies would ensure the specific detection of the harmful food content for a sensitised person.

The production of functional antibody fragments and their efficient display on the filamentous phage have made it possible to construct large and diverse antibody phage display libraries for the isolation of recombinant antibodies. Aim of the study was to construct mouse IgG antibody libraries from immunised mice. The selection of the resulting libraries was carried out using recombinant allergens, rMal d 1 and rApi g 1 which were produced as soluble non-fusion proteins in bacterial cells and purified by a conventional chromatography. The binding properties of the isolated Fab fragments towards the recombinant allergens and native allergens from natural sources were characterised. Interestingly, isolated Mal d 1 -specific antibody bound also to Bet v 1, the main allergen eliciting the cross-reactivity syndrome between the birch pollen and apple. Despite the similarities in protein tertiary structure, there was no observable cross-reactivity between Api g 1 -specific antibodies and Bet v 1 in this study. Isolated allergen-specific antibodies can be utilised as diagnostic and research tools for the specific and reliable detection of allergens from different consumable products as well as studying allergy-antibody interactions.

